# Our Book Table

**Published:** 1885-01

**Authors:** 


### OUR BOOK TABLE.

THE FORMATION OF POISONS BY MICRO-ORGANISMS. *A Biological Study of the Germ Theory of Disease.* BY G. V. BLACK, M. D., D. D. S. Philadelphia: P. Blakiston, Son & Co., 1884.

Immediately upon the reception of this work we gave it due notice, but the time was insufficient for such careful reading as would permit the formation of an intelligent opinion of its merits.

The first and second parts of the book are devoted to a general review of the whole subject, and to a consideration of the theories advanced and the work accomplished by the eminent biologists who have made a special study of the matter. In condensed form is presented the gradual growth of the bacterian hypothesis, and the conclusions of men like Schwan, Schroeder, Bastian, Beale, Pasteur, Koch, and others.

The appendix is devoted to a consideration of the Germ Theory of Disease in its Relation to Dental Caries. The author reviews the work of Leber and Rottenstein, and of Magitot, and the theories of Watt, but bestows the greatest amount of attention upon the later work of Miller. He says that the latter commences where Magitot left off sixteen years previously, and that during that interregnum no new facts of importance had been developed or discovered. He traces the line of experiments of Dr. Miller, admits the important points so incontestably demonstrated by him, and states that the isolating of one or more acid-producing fungi, the preparation of pure cultures of them, and by their influence the successful decomposition of sound human dentine, "when compared with the best experimentation previously had marks a great advance."

The author then proceeds to draw attention to what he thinks may prove another source of caries of teeth. He says: "I have already spoken of the strong probability that the otherwise normal tissues, when under the influence of certain qualities of inflammation, emit a fluid of acrid and very irritating properties. * * * If a piece of ivory thrust into the flesh is attached and burrowed out in holes by a secretion thrown out by virtue of the irritation induced, as asserted by Krause, Kölliker, and others of the most capable observers of the world, why may not a tooth be attached in the same way, by virtue of the irritation of the tissues about its neck, or during the irritation consequent upon its eruption when this is unusually prolonged?"

This is worthy our serious consideration, yet we are afraid that the author will not find that dental pathologists will generally accept it as a primary cause of dental decay. At most it can be but secondary, for there is a state antecedent to this effusion that is pathological of itself. According to Dr. Black's own statement it is the result of irritation, and this then would be the prime factor, and a cure would be sought, not in neutralizing this effusion, but in removing the offending cause of it. If the bacterian origin of most dental decay be admitted, as it is by Dr. Black, and Dr. Miller's established facts that the micro-organisms secrete or excrete an acid that is capable of disorganizing tooth structures, why should not these very fungi, as Dr. Miller asserts, be the origin of this acrid fluid, and thus the primal cause of the species of decay of which Dr. Black speaks? If we admit the formation of an acid or an acrid fluid by fungi that shall be sufficient for the production of decay in one case, why is it not most consistent to at least look for it in all analogous instances? The very title of the book is "The Formation of Poisons (acrid or irritating fluids?) by Micro-Organisms." It would, therefore, seem as if the cause assigned by the author as primary might readily be traced to an antecedent one, not very far removed.

The book is the latest and best of the reviews of the bacterian theory of disease from the standpoint of the dentist, and as such, aside from the respect which every one must feel for the opinions and deductions of so thorough a student, so competent an observer, so learned a writer and teacher, and so candid a man as is Dr. Black, it will command the attention of every intelligent dentist, and we hope will be widely read and thoroughly studied, as it deserves to be.

A Complete Index to the Dental Cosmos. The S. S. White Dental Manufacturing Co., Philadelphia, 1884.

No dental library is complete without a full set of the *Dental Cosmos*, and certainly no one can obtain the full benefit of this encyclopedia of dental science without the complete index to the twenty-four volumes already issued. The book owes its existence to the continual need for it felt by Dr. A. L. Northrup, who began the work afterward carried to completion by Dr. Jas. E. Dexter. Some idea of the comprehensiveness of the volume and of the amount of labor bestowed upon it may be gathered from a knowledge of the fact that it contains 30,000 references. Every topic that is treated of anywhere in the twenty-four volumes is appropriately indexed under the several headings of authors, titles, subjects. For instance: "Anæsthetics" has 260 references, and other important subjects have a proportional number. The references to authors form a curious subject of study, and indicate how thorough has been the scanning of all the volumes. Naturally, the editors of the journal come in for the greatest number of quotations, and we find that Dr.

J. H. McQuillen is referred to 217 times in the first thirteen volumes, while Dr. James W. White is credited with 232 references in the last twelve. Dr. James D. White has 140 references in the first fourteen volumes. Dr. J. F. Flagg leads the list of contributors with 134 quotations, and he is followed by Dr. Garretson with 98, Dr. Atkinson with 77, and Dr. Latimer with 45. There have been published in the *Dental Cosmos* 267 reviews, more or less elaborate, of new books and pamphlets relating to dentistry. Of "recipes" 630 have been given, and each is so thoroughly tabulated that it may readily be found. Under the head of "Tumors" there are 188 references.

We might go on speaking of the interesting facts revealed by the volume, the evidences of the immense amount of labor involved in the editing of a first-class professional journal, but we can only do it effectually by referring the curious to the volume itself.

MEDICAL RHYMES. *A Collection of Rhymes of ye Ancient Time, and Rhymes of the Modern Day; Rhymes Grave and Rhymes Mirthful; Rhymes Anatomical, Therapeutical and Surgical; All Sorts of Rhymes to Interest, Amuse and Edify all Sorts of Followers of Esculapius.* Selected and compiled from various sources by HUGO ERICHSEN, M. D., with an introduction by PROF. WILLIS P. KING, M. D. Illustrated: J. H. Chambers & Co., St. Louis, Mo., 1884.

This handsome volume of 220 pages is just the thing to amuse the leisure hours of a physician. Its scope and character are amply set forth in the full title which we have quoted. It must not be thought, however, that it is a collection of jingling doggerel, for it is far removed from this. Some of the poems are by famous authors, and are a part of the standing literature of the age. Some of them contain the most dainty bits of sentiment. Some are very humorous, while others are almost lachrymose in their solemnity. But the selections are most admirable in their character, and together they form a volume that would prove a tasteful ornament to the reception room of any general practitioner, or medical specialist. J. H. Chambers & Co. are gaining the reputation of furnishing to the profession dainty volumes, and this is one of their handsomest.

A NEW METHOD OF RECORDING THE MOTIONS OF THE SOFT PALATE. BY HARRISON ALLEN, M. D., Professor of Physiology in the University of Pennsylvania. Philadelphia: P. Blakiston, Son & Co., 1884.

This is a most ingenious monograph of a very ingenious method of determining the exact motion of the soft palate in talking, coughing, inhaling, exhaling, swallowing, etc., etc. A straight rod passed through the nose from before backward in the living subject as far as possible, impinges against the roof of the naso-pharynx. The external end of the rod now being raised, the pharyngeal end lies in such a position as that it will be influenced by every motion of the soft palate. By an ingenious contrivance this is so connected with a revolving barrel that sphygmographic tracings are made upon prepared paper. The sounds of the various letters are now given, sentences repeated, and all kinds of articulate noises made, which are instantly recorded. The tracings of many of these are given in the book, and some curious facts revealed. It is impossible within the limits of such a notice as this properly to analyze them, but it is evident that their study may greatly assist the analysis of language, and perhaps aid in the better understanding and cure of stammering, and other infirmities of speech.

We have also received the following books and pamphlets:

*Transactions of the Michigan State Dental Society*, 1884.

*Transactions of the Louisiana State Medical Society*, 1884.

*Notes on the Opium Habit.* Third Edition. Revised and enlarged. By Asa P. Meylert, M. D. New York: G. P. Putnam's Sons.

*Muriate of Cocaine in Ophthalmic Surgery.* By C. J. Lundy, M. D. Reprinted from *The Physician and Surgeon.*

*New and Improved Process of Manipulating Gold, Rubber, and Celluloid, for Dental Purposes.* By Frederick Wheaton Seabury, Dentist, Providence, R. I.

*The Rubber Question.* By Mrs. M. W. J. Reprinted from the *Southern Dental Journal.*

*Oral Hygiene.* By Meyer L. Rhein, M. D., D. D. S. Reprinted from the *New England Journal of Dentistry.*

*One Aspect of the Subject of Medical Examination.* Report of the Secretary of the North Carolina Board of Health.

*Jequirity: Its Uses in Diseases of the Skin.* By Jno. V. Shoemaker, A. M., M. D.

*Notes on the Treatment of Trachoma by Jequirity.* By Leartus Connor, A. M., M. D.

*Mumps as a Cause of Sudden Deafness.* By Leartus Connor, A. M., M. D.

*The Dry Treatment of Chronic Suppurative Inflammation of the Middle Ear.* By Charles J. Lundy, A. M., M. D.

*Fleer & Company's Dental Catalogue,* 1884.

*Bericht ueber die Verhandlungen der VI. Jahresversammlung der Zahnarzte Skandinaviens.* Separat-Abdruck aus dem "*Correspondenz Blatt fuer Zahnaerzte.*"

*Die Ausuebung der Zahnheilkunde im deutschen Reiche.* Separat-Abdruck aus der *Koelnichen Zeitvng.*

